# Radiographical presentations of inadvertent subdural placement of an epidural catheter for successful labor analgesia: A case report

**DOI:** 10.1097/MD.0000000000036000

**Published:** 2023-12-01

**Authors:** Wei-An Chen, Ming-Cheng Wu, Chen-Fuh Lam, Chang-Hsien Ou

**Affiliations:** a Department of Anesthesiology, E-Da Hospital and I-Shou University, Kaohsiung, Taiwan; b Department of Anesthesiology, Dalian Tzu Chi Hospital, Buddhist Tzu Chi Medical Foundation, Chia-Yi, Taiwan; c Department of Neuroradiology, E-Da Hospital and I-Shou University, Kaohsiung, Taiwan.

**Keywords:** case report, computed tomography, epidural analgesia, myelography, painless labor, patient-controlled epidural analgesia, subdural analgesia

## Abstract

**Rationale:**

Lumbar epidural analgesia is the gold standard for labor pain control. However, misplacement of epidural catheters into the subdural space may inadvertently happen. Unrecognized subdural administration of local anesthetics could result in serious consequences, including high spinal and brainstem blocks. This case report describes a case where subdural epidural catheter placement was recognized early but labor pain was adequately managed by dosage titration of subdural analgesia.

**Patient concerns:**

This case report describes a 29-year-old primiparous pregnant woman who was admitted to our obstetric unit for labor induction at the gestational age of 38 weeks. An epidural catheter was inserted via the L2-3 intervertebral space using the standard loss of resistance to air technique.

**Diagnoses:**

The parturient experienced weakness in the lower extremities and numbness in the upper extremities within 15 minutes after administration of 5 mL of 2% v/v lidocaine as a loading dose and systolic blood pressure also dropped by 25%.

**Interventions:**

The dose regimen (a mixture of 0.1% ropivacaine and 4 μg/mL fentanyl) for patient-controlled analgesia was given with bolus doses of 0.1 mL per demand and lockout intervals of 20 minutes. The analgesic effects were adequately maintained below the T8 dermatome for more than 12 hours without hypotensive episodes or obvious signs of neurological deficits. Computed tomographic myelography was performed by instillation of a nonionic iodinated contrast medium via the epidural catheter on postpartum day 2 for imaging confirmation of catheter placement in the extradural space.

**Lessons:**

Early recognition that epidural catheters for neuraxial analgesia have been inserted into the subdural space is important for the prevention of high spinal blocks. Subdural analgesia could still be achieved by careful clinical assessment and titration of low analgesic doses. This report also presents important and clear serial computed tomographic images of catheter placement in the thoracic-lumbar subdural spaces and the extent of volume spread in the subdural space following administration of contrast medium.

## 1. Introduction

Patient-controlled epidural analgesia (PCEA) is recommended as the gold standard for analgesia management in labor pain and up to 60% of parturient women in developed countries use PCEA for painless labor.^[1]^ To place an epidural catheter, an epidural needle is introduced through the supra- and interspinous ligaments, passing through the ligamentum flavum. The loss of resistance technique is usually used to confirm needle tip placement in the epidural space. The epidural catheter is then threaded into the epidural space for the delivery of analgesic agents. However, the loss of resistance technique can sometimes result in the epidural catheter being threaded into the subdural space, as the subdural space is a virtual anatomical space occupied by serous fluid adjacent to the epidural space.^[2]^ The clinical presentations of unintentional subdural blocks in the thoracic-lumbar regions can be variable and the responses depend on the extent of the spread of local anesthetics.^[3]^ As the subdural space has limited capacity for volume spread, unexpected high neuraxial blocks usually develops with 15 to 20 minutes after drug administration and can even result in cranial nerve paralysis and profound hypotension.^[3]^ Therefore, the misplaced catheter in the subdural space is usually recommended to remove and reinserted into the epidural space if indicated.^[3]^

According to large-scale retrospective studies, the incidence of inadvertent subdural catheter placement is approximately 0.53% to 0.82% during epidural catheterization.^[3,4]^ In a prospective study of 145,550 cases involving obstetric epidural analgesia, the incidence of subdural injections was 0.024%.^[5]^ However, most subdural blocks were identified based on clinical findings and without radiographical evidence.

This case report describes a case where the subdural placement of an epidural catheter was recognized early following administration of a loading dose of local anesthetic in a parturient, and the inadvertently inserted subdural catheter was used for labor pain management through careful clinical assessment and titration of PCEA dose regimen. Most significantly, we present a series of contrast-instilled computed tomography (CT) myelographic images via epidural catheter to identify the catheter track and volume spread of contrast medium in the subdural space.

## 2. Case report

A 29-year-old primiparous pregnant woman (body height of 142 cm) was admitted to our obstetric unit for labor induction at the gestational age of 38 weeks. She denied having any significant medical history and did not have previous surgical history. PCEA was arranged for management of labor pain during the induction period. An epidural needle (18G, Weiss epidural needle, BD) was inserted at the L2-3 intervertebral space in the lateral decubital position and the needle tip was advanced until the occurrence of loss of resistance to air in the epidural syringe (Epilor plastic LOR syringe, BD). An epidural catheter (20G, Perisafe Nylon Catheter, BD) was threaded into the low resistance space and the catheter was secured at the 11-cm mark on the inserted site.

After the parturient was returned to the normal supine position, 5 mL of 2 v/v% lidocaine was injected via the epidural catheter as a loading dose for analgesia. About 15 minutes later, the parturient started to experience bilateral motor weakness in the lower extremities and bilateral numbness in the upper extremities. At the same time, the patient’s systemic blood pressure dropped to 88/50 mm Hg, but there were no other neurological signs or further hemodynamic changes. Signs of upper limb numbness gradually regressed over the next 10 minutes. Another bolus of 1 mL of 2 v/v% lidocaine was injected via the epidural catheter, and we identified that the sensory block levels were confined below the T8 dermatome. Although subdural placement of the catheter was highly suspected, sufficient sensory block was achieved through reduced doses of local anesthetic. We thus opted to use the catheter for managing labor pain by adjusting the analgesic dose regimen. The patient-controlled dose of analgesics was titrated to 0.5 mL bolus-only regimen containing 0.1% ropivacaine and 4 μg/mL fentanyl with lockout intervals of 20 minutes. Levels of neuraxial block were carefully assessed at 1-to 2-hours intervals. Hemodynamics and the respiratory rate of the parturient were closely monitored and cardiotocography was also applied for continuous monitoring of the fetal heart rate. Labor pain was adequately controlled to maintain visual analogue scales <3 throughout the active labor period. However, the parturient received a cesarean section under spinal anesthesia about 12 hours after labor induction due to failure of labor progression.

**Figure 1.**
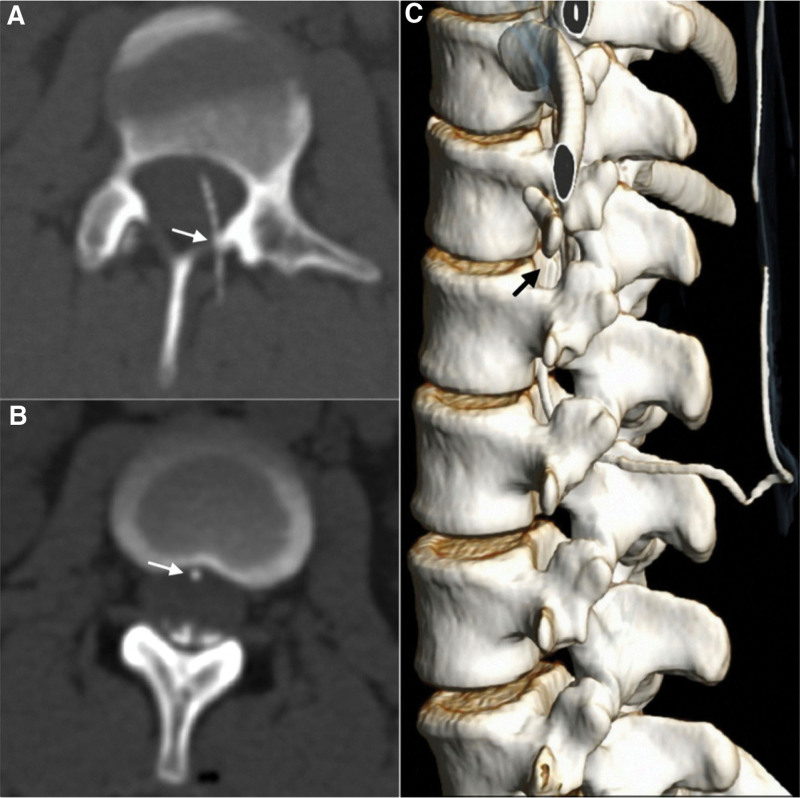
Computed tomographic myelography of thoracolumbar spine following instillation of contrast medium via the epidural catheter. (A) Axial image with MIP of the lumbar spine at L2 level showing the entry point (arrow) of the epidural catheter and the catheter traveling across the dorsal to the ventral portion of within the dural sac. (B) Axial image of the lumbar spine at L1-2 intervertebral disc level demonstrating the location of epidural catheter in the ventral site of the dural sac (arrow) and contrast medium collection in the dependent portion of the dural sac. (C) 3D reconstruction computed tomographic image demonstrated the virtual anatomy of the spine and the whole route of the epidural catheter in the subdural space. The tip of the epidural catheter was located at the T12-L1 disc level (black arrow). MIP = maximum intensity projection.

**Figure 2.**
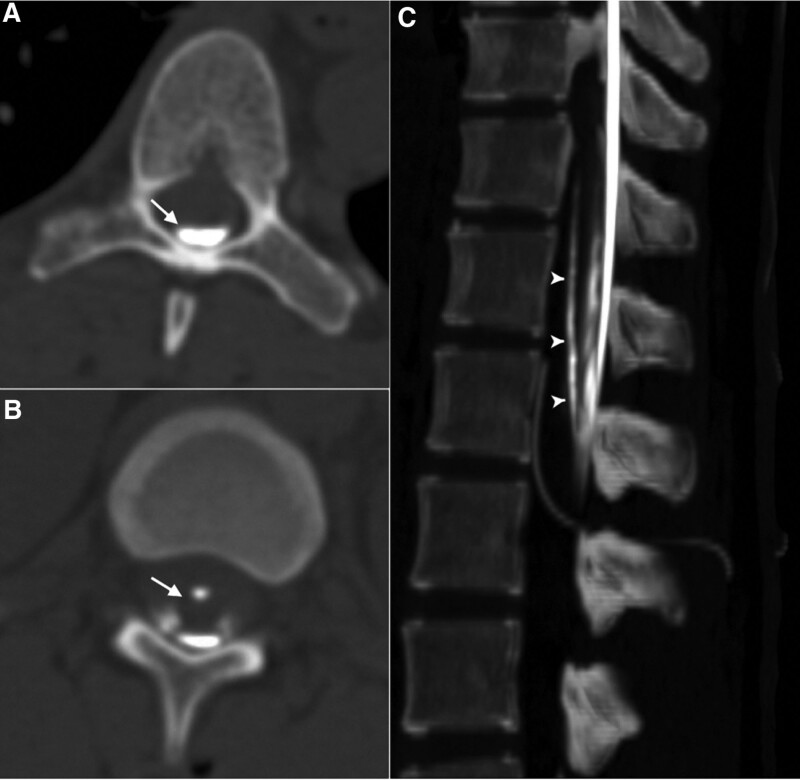
Computed tomographic myelography of thoracolumbar spine following instillation of contrast medium via the epidural catheter. (A) Axial section of the T9 level demonstrated pooling of contrast medium within the dependent site of dural sac (arrows). (B) Axial section at T12 level demonstrated accumulating of contrast medium in the dependent part of the dural sac and surrounding the conus medullaris (arrow). (C) Sagittal reconstructed maximum intensity projection (MIP) image clearly demonstrated the whole route of the epidural catheter entering the intradural space. Instilled contrast medium spreads in the dependent portions of subdural space from T8 to L2 levels. The distribution of the contrast medium in the subdural space was presented with the typical “railroad track” appearance (arrowheads).

On postpartum day 2, a water-soluble nonionic iodinated contrast was instilled via the epidural catheter during CT myelographic examination to confirm the exact placement of the epidural catheter in the meningeal planes. A total of 1.5 mL contrast medium was injected into the epidural catheter while the patient was in the neutral supine position. The CT images with maximum intensity projection and 3-dimensional reconstruction showed that the catheter was entered into the subdural space at the L2 level and traveled upward in the subdural space across from the dorsal site to the ventral site at the T12 level (Fig. 1A–C). The tip of the epidural catheter was located at the T12-L1 disc level (Fig. 1C). The contrast medium spread in the dependent portions of subdural space from L2 to T8 level (Fig. 2A–C) and was presented with the typical “railroad track” distribution (Fig. 2C).^[4]^

The epidural catheter was removed after the CT scan. The postpartum woman was discharged at postpartum day 4 and no neurologic deficits were reported up to 1 week after being discharged.

## 3. Discussion

Several cases where inadvertent subdural catheters have been successfully used in cesarean sections for neuraxial anesthesia have been reported.^[2,6]^ However, the use of subdural analgesia for painless labor with patient-controlled bolus-only regimen has not been previously reported in the literature. We also present definite radiographic evidence for subdural analgesia in this study.

Since the spinal nerves in the subdural space are enveloped within the pia and arachnoid maters, spinal nerve block onset is often relatively slow (15–20 minutes after injection of local anesthetics).^[3]^ In addition, the very limited volume capacity of the subdural space results in disproportionately high dermatomal neuraxial block levels following subdural injection of local anesthetics.^[3]^ In the case described above, the presence of the 2 aforementioned characteristic responses after local anesthetic injection strongly suggested that the epidural catheter was unintentionally inserted into the subdural space. We further examined the sensory block levels by administering an even smaller volume of lidocaine and found that the levels of sensory block were confined to the regions for adequate control of the active labor period. To avoid further neuraxial block at higher dermatomal levels, the patient-controlled bolus dose was titrated to 0.5 mL per demand with a lockout interval of 20 minutes and the continuous infusion dose was turned off. The labor pain was then effectively controlled for up to 12 hours and potential adverse effects were carefully monitored throughout the course.

The other clinical importance of this study is the radiological studies (plain, maximum intensity projection and 3D reconstructed CT images) that identified the exact location of the epidural catheter and the regional spread volume in the meningeal spaces. The radiographical findings were consistent with a previous report that the catheter injection of contrast medium would maintain a dependent position without anterior extension and the presence of a typical “railroad track” distribution of contract medium due to cerebrospinal fluid dilution in the subarachnoid space.^[4]^

In conclusion, inadvertent subdural catheter placement may occur during epidural catheterization using the standard loss of resistance to air technique. With early recognition and careful assessment, subdural analgesia may still be feasible for managing labor pain through titration of the anesthetic dose. However, we do not recommend the use of subdural analgesia in the presence high neuraxial blocks or inadequate sensory block levels after administration of the second low-titration dose of local anesthetics.

## Author contributions

**Conceptualization:** Wei-An Chen, Ming-Cheng We, Chang-Hsien Ou.

**Data curation:** Wei-An Chen, Chang-Hsien Ou.

**Formal analysis:** Chen-Fuh Lam.

**Investigation:** Chen-Fuh Lam.

**Software:** Chang-Hsien Ou.

**Supervision:** Chang-Hsien Ou.

**Validation:** Chang-Hsien Ou.

**Writing – original draft:** Wei-An Chen, Ming-Cheng We.

**Writing – review & editing:** Chen-Fuh Lam, Chang-Hsien Ou.
